# Acute Spinal Cord Contusion in a Patient with Multiple Upper Cervical Fractures, Parkinson's Disease, and Torticollis: Surgical Management

**DOI:** 10.1155/2020/8897071

**Published:** 2020-09-11

**Authors:** Sarah Merrill, Maziyar A. Kalani, Naresh P. Patel, Mark K. Lyons, Matthew T. Neal

**Affiliations:** Mayo Clinic Arizona, Department of Neurological Surgery, 5777 East Mayo Boulevard, Phoenix, AZ 85054, USA

## Abstract

*Case Report*. Spine surgery in patients with Parkinson's disease (PD) involves increased risk. We describe a case of cervical myelopathy in a patient with PD, multiple fractures involving the atlas and axis vertebrae, and spasmodic torticollis. The patient was successfully treated with an upper cervical decompression and occipital-cervical (OC) fusion surgery. Strategies for torticollis reduction and successful surgical outcome are discussed. Risks and benefits must be carefully weighed when considering occipital cervical fusion in PD patients. *Conclusion*. Intraoperative manual reduction of laterocollis is possible after general endotracheal anesthesia, and continuous neuromonitoring is established. Use of optimizing strategies such as perioperative botulinum injections and intraoperative O-arm navigation should be considered.

## 1. Introduction

Parkinson's disease (PD) is a relatively common neurological disease, affecting roughly 1% of the population over 60 years old [1]. The typical clinical presentation of PD includes tremor, rigidity, bradykinesia, and postural instability [2]. PD is also often associated with nonmotor symptoms including fatigue, sensory abnormalities, autonomic dysfunction, and psychological and behavioral changes [3]. Patients with PD are at greater risk of falls and fractures, and risk of injury is compounded by the high rates of osteoporosis [4–6]. Associated neuromuscular changes from focal myopathy to dystonia can contribute to the development of spinal malalignment issues in multiple planes [7–9]. These comorbidities are important considerations when selecting an appropriate treatment approach. This case describes a unique surgical challenge in a PD patient with multiple cervical fractures, compromised bone quality, and spasmodic torticollis. To the authors' knowledge, no similar case has previously been described. Surgical techniques and optimization strategies for this patient are discussed.

## 2. Case Report

**Figure 1:**
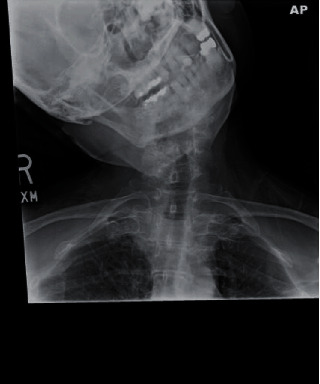
Preoperative cervical X-ray demonstrating severe right laterocollis.

We report the case of a highly functional 76-year-old female with a history of osteoporosis and well-controlled idiopathic PD being treated with pramipexole and Azilect. Four months prior to presentation, she suffered a fall in the garden resulting in intractable upper cervical pain. The pain was positional and alleviated with resting supine. Physical therapy and numerous pain medications failed to result in any significant pain reduction. Shortly before presentation, she suffered severe worsening of her right laterocollis (Figure 1). In addition, she developed progressive myelopathy including bowel and bladder incontinence, right-sided weakness, and loss of independent ambulatory status.

Her examination was remarkable for severe right laterocollis. The laterocollis was preexisting and not related to the acute injury but became worse following the injury. The severity of the cervical pain resulted in the patient mechanically altering her cervical posture and exacerbating the laterocollis due to progressive cervical spasm. She was unable to voluntarily correct the posture. Efforts to reduce the laterocollis manually resulted in severe pain. She was confined to a wheelchair, and she had 4+/5 strength in right-sided extremities. She had mild cogwheel rigidity in all extremities and bradykinesia in hand movements, in addition to diffuse hyperreflexia in her extremities.

**Figure 2:**
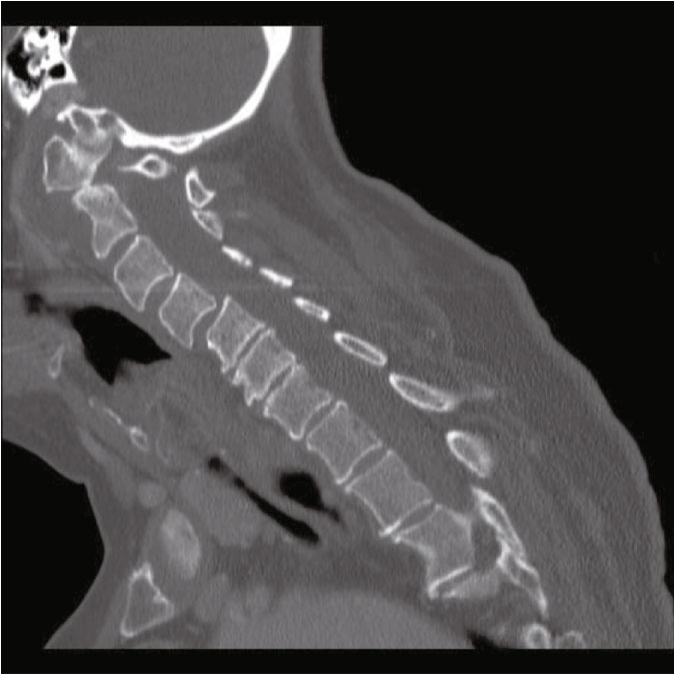
Cervical CT showing subluxation of the posterior C1 ring into the spinal canal.

**Figure 3:**
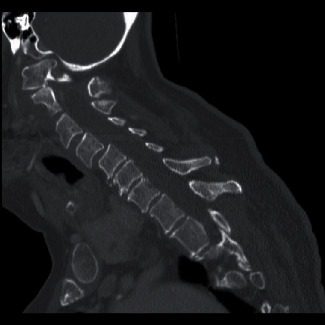
Cervical CT showing 1 of the multiple C1 fractures.

**Figure 4:**
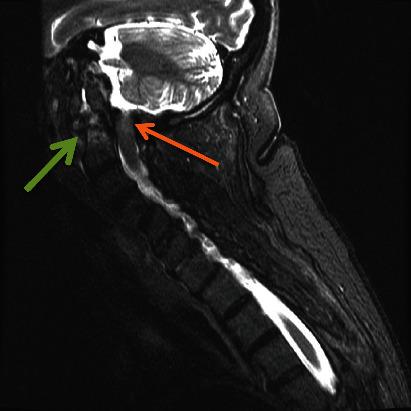
Cervical MRI (STIR) showing increased STIR signal in the spinal cord at the C1 level (orange arrow) and odontoid fracture (green arrow).

Imaging revealed subluxation of C1 on the C2 vertebra (Figure 2) and loss of structural integrity due to subacute comminuted C1 fractures and a type 2 odontoid fracture (Figure 3). She had severe stenosis at C1 due to the subluxation of the posterior C1 ring into the spinal canal. There were severe cord compression and cord signal change at this level (Figure 4). She also had diffuse spondylosis, kyphosis, and chronic anterolisthesis of C4 on C5.

A frank discussion was held with the patient and family regarding the surgical risks. The risks of hardware failure in setting of her severe kyphosis, osteoporosis, and severe cervical dystonia were discussed. Different surgical strategies and constructs were considered by the surgical team. To minimize morbidity and address the myelopathy, we planned a focal operation from the occiput to C3.

To reduce her cervical dystonia, botulinum toxin (Botox®) injections in the sternocleidomastoid, splenius capitis, trapezius, and scalene muscles were given one week prior to surgery. Intraoperatively, the patient was placed under general anesthesia without muscle paralysis. Baseline motor evoked potentials (MEPs) and somatosensory evoked potentials (SEPs) were performed. The patient's head was secured in the Mayfield head clamp in her natural position. MEPs and SEPs were run again and remained stable. Over 30 minutes, we gradually reduced her torticollis and resecured her head and neck in neutral alignment for surgery. Neuromonitoring potentials remained stable and unchanged during the manipulation. The occiput and upper cervical spine were exposed using a standard surgical technique. Computer tomography- (CT-) based 3-dimensional navigation (O-arm ®) was used for the precise placement of the occipital plate screws, C2 pedicle screws, and C3 lateral mass screws. We then performed C1 and C2 laminectomies to decompress the spinal cord. Intraoperative navigation was extremely valuable for safe exposure and resection of the C1 laminar fragment due to the overlying soft tissue pannus and depth of the fragment within the spinal canal. Bilateral rods were secured to the plate and screws. Arthrodesis was performed from the occiput through C3 using locally harvested morselized autograft and demineralized bone matrix putty (DBM).

**Figure 5:**
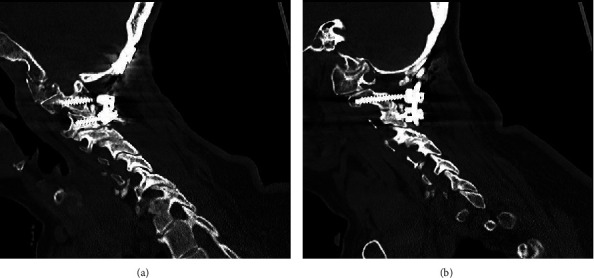
(a, b) Cervical CT one year after surgery demonstrating the occipital-C3 construct.

**Figure 6:**
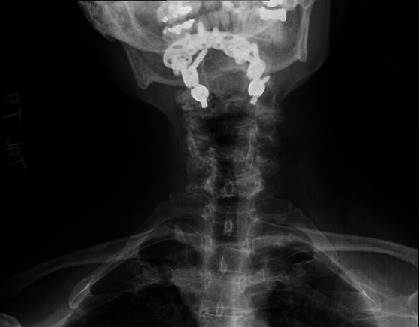
Postoperative AP cervical X-ray showing improvement in lateral deviation of the head following fusion.

One year following surgery, the patient's myelopathy symptoms were markedly improved. She recovered full strength on her right side and was able to ambulate without the assistance of a walking aid. Her CT one year after surgery showed robust fusion from the skull base through C3 (Figure 5). Her X-rays (Figure 6) showed significant improvement in the lateral posture of her neck compared to her preoperative condition.

## 3. Discussion

The patient in this case was a high-risk surgical candidate, and the decision to proceed with surgery was carefully calculated. PD patients have been shown to have higher complication rates and nonhome discharges following cervical surgery compared to patients without PD [10]. Biomechanical challenges from sagittal malalignment of the spine [11] and osteoporosis [4, 6, 12] contribute to the higher complication rates in PD patients. The patient in this case had prominent cervical kyphosis and osteoporosis.

Retrospective clinical studies have shown long-term hardware complication rates following occipital-fusion surgery of approximately 30% [13, 14]. Increased reoperation rates in patients older than 65 years following occipital cervical fusion have also been reported [15]. Another complicating factor was the patient's laterocollis. It was unclear if the patient's powerful muscular spasms would dislodge the hardware postoperatively.

Unfortunately, the patient had experienced significant progressive neurological deterioration. Retrospective studies have shown high rates of symptomatic improvement, in excess of 90%, following occipital cervical fusion [16, 17]. The majority of patients with myelopathic symptoms such as gait or functional deficits improved following occipital cervical fusion surgery [13, 14, 16]. Deutsch et al. found that 30% of patients reported their myelopathic symptoms improved dramatically (one Nurick grade) following occipital cervical fusion surgery [14]. Given the possibility for symptomatic improvement and lack of effective nonsurgical alternatives, the patient elected to proceed with surgery.

Cautious surgical planning including use of perioperative botulinum injections and intraoperative O-arm-navigated hardware placement was critical for success in this case. Perioperative use of botulinum toxin injections has previously been reported as an adjunct for cervical surgery [18, 19]. In this case, botulinum toxin was injected into the sternocleidomastoid, splenius capitis, trapezius, and scalene muscles 1 week before and approximately 4 weeks after surgery. The preoperative injections facilitated the intraoperative manual reduction of the laterocollis, and the postoperative injections aided in spine immobilization following surgery. The use of spinal navigation has been shown to increase accuracy and decrease complication rates when placing OC hardware in particular with C2 screw placement [20, 21]. We conclude that the use of CT-guided 3-dimensional navigation was critical for the precise placement of the hardware including the midline screws in the occipital keel and pedicle screws in C2. Navigation also allowed us to maximize screw length and purchase in the osteoporotic bone.

## 4. Conclusion

Risks and benefits must be carefully weighed when considering occipital cervical fusion in PD patients, given the multitude of potential comorbidities that may complicate the perioperative recovery. Safe manual reduction of laterocollis, even with upper cervical fractures, is possible with the aid of general anesthesia and continuous neuromonitoring. The use of perioperative botulinum injections and intraoperative O-arm navigation was a critical strategy in this case allowing reduction of torticollis, safe spinal cord decompression, and robust spinal fixation.

## Data Availability

Readers can access the data supporting the conclusions of the study via the references in the manuscript that support the conclusions of the paper.
